# DNA methylation in senescence, aging and cancer

**DOI:** 10.18632/oncoscience.476

**Published:** 2019-01-31

**Authors:** Wenbing Xie, Stephen B. Baylin, Hariharan Easwaran

**Affiliations:** The Sidney Kimmel Comprehensive Cancer Center at Johns Hopkins, The Johns Hopkins University School of Medicine, Baltimore, MD 21287, USA

**Keywords:** DNA methylation, senescence, aging, promoter CpG-island, epigenetic

In response to genotoxic stresses, cells are programmed to trigger senescence, involving metabolic slowdown and proliferation arrest, to contain deleterious effects of the damaged genetic material [1]. Two forms of senescence processes are relevant in this context: (a) Replicative Senescence (RS) in response to telomere shortening and/or reactive oxygen species (ROS) that occur during aging; (b) Oncogene Induced Senescence (OIS) in response to oncogenic mutations, such as oncogenic RAS mutations. Failure to trigger senescence can lead to tumorigenesis. Epigenetic alterations play important roles during both senescence and tumor initiation. During senescence, epigenetic alterations play important roles in stably silencing proliferation-promoting genes [2], while in tumorigenesis the epigenetic changes function in suppressing tumor suppressor genes. Here we summarize recent advances in understanding the relation and origins of senescence and tumor epigenomes, and its implications for tumorigenesis.

## Replicative senescent and cancer epigenomes – similar but yet different

Global CpG methylation losses and focal gains at promoter CpG islands (CpGI), are a hallmark of cancers [3]. Similar global hypomethylation and CpGI hypermethylation are also observed in RS. A key hypothesis from these observations is that tumor-promoting DNA methylation in cancers may stem from cells escaping RS, and that DNA methylation patterns in RS may promote tumorigenesis once RS is bypassed [4]. To explore this, we analyzed in detail genome-wide gene expression and DNA methylation patterns in Weinberg's classical transformation system [5], i.e. the immortalization and transformation stages involving sequential introduction of human telomerase reverse transcriptase (hTERT), simian virus 40 large T antigen (SV40) and oncogenic HRAS^G12V^. In parallel, similar analyses were performed in early stages of RS (near-senescent) and RS cells [5]. Both transformed and RS cells harbor global losses and focal CpGI gains of DNA methylation, consistent with previous observations [4]. However, the individual genomic regions involved in the two processes are strikingly different and phyloepigenetic analyses revealed a stochastic accumulation of methylation changes in the immortalization-transformation processes, whereas RS involves programmed methylation alterations. Promoter CpGI methylation gains in transformation mainly target development and differentiation genes, while those in near-senescent and senescent states primarily involved in genes positively regulating biosynthetic and metabolic processes. Thus, hypermethylation-mediated silencing, or more importantly loss of induction, of developmental genes during transformation, may facilitate cancer cell self-renewal and survival. In contrast, silencing the biosynthetic and metabolic genes in RS could be necessary for slowing the metabolism of cells undergoing RS. Interestingly, a subset of hypermethylated genes overlapped between the two processes. These commonly methylated genes were also significantly enriched for developmental regulators and some, such as *CDH1*, *SOX17* and *MGMT*, are important tumor suppressor genes frequently methylated and silenced in various human cancers. Whether such methylation events during early stages of RS provide some proliferative potential for few cycles of cell divisions, until complete shutdown of the cell cycle and metabolic machinery takes effect, remains a plausible scenario that needs to be explored.

Considering all the above findings, another important question is whether DNA methylation patterns in senescent cells may promote tumor formation if RS is bypassed. Introduction of hTERT, SV40 and HRAS^G12V^ into proliferating near-senescent cells, which already harbor the RS-associated methylation patterns, results in immortalization but not transformation. Thus, RS-associated DNA methylation may contribute to resistance against transformation. Our studies thus provide an important context to previous studies and suggest that the tumor epigenome may not be derived from the senescent associated epigenetic changes.

## Origin of cancer epigenome from cycling aging cells

Aging is the biggest cancer risk factor and senescence is well characterized to be related to aging [1]. And genes with promoter hypermethylation in cancers have been shown to acquire methylation during aging [6]. Therefore, we related three groups of genes we identified: HSM, HRAS (transformation)-specific methylated genes; SSM, replicative senescence-specific methylated genes; CM, commonly methylated genes, to aging. We found that HSM and CM hypermethylated developmental genes, but not the SSM associated-metabolism genes, exhibit significantly higher methylation gains in colon and lung primary tumors compared with normal tissues. Promoters of HSM and CM genes also have increased likelihood of hypermethylation during aging of various normal tissues wherein the gains in methylation track the age-associated risk of cancer. Therefore, transformation associated genes, not necessarily senescence-associated genes, are hotspots for DNA hypermethylation not only in primary tumors but also in aging tissues. Thus cancer cells and the associated methylation patterns most likely evolve from proliferating, aging cells accumulating stochastic methylation but unlikely from growth-arrested senescent cells. Importantly, the above genes may thus be important for tracking age-associated cancer risk.

## Modes of senescence and senescence reversal in cancers

A key finding from our studies is that the near-senescent cell state, which is the precursor to RS, could not be transformed, and that the RS-epigenome may have a role in this process. Interestingly, we showed that OIS, which occurs acutely in a short duration (10 days), does not accompany any DNA methylation alterations. In OIS, histone H3 lysine 9 (H3K9) mediated inactivation of proliferation genes has an important role [2]. Recently Yu et al. studied reversal of OIS in melanocytic nevi, and they showed that activation of lysine-specific demethylase-1 (LSD1) and several Jumonji C domain-containing moieties (such as JMJD2C) caused reversal of OIS and allowed Ras/Braf-induced transformation [7]. This study showed that the OIS state could be restored in human melanoma samples by inhibiting the H3K9 demethylase activity. The studies by Yu et al. highlight the importance of modulating the epigenome for reversing senescent to cancer state, and vice versa, which have huge implications for cancer prevention and treatment. These studies also raise the important question for RS – whether reversing DNA methylation alone or in combination with other inactivating histone marks can reverse RS.

In another important study, it was shown that the mode of senescence induction and the cellular context has important consequences for cancer biology and management. Milanovic et al. showed that chemotherapy induced senescence in acute lymphoblastic leukemia and acute myeloid leukemia mouse models, as well as in human hematological malignancies, caused conversion of non-stem leukemia cells to self-renewing, leukemia initiating cells [8]. The senescence-induced stemness in already transformed leukemic cells thus causes aggressive relapse of tumors. The study by Milanovic et al. raises important questions about the epigenetic reprogramming process and its tumor protective or preventive properties in the context of studies by us and Yu et al. A key concept to comprehend is that senescence associated epigenetic reprogramming has very different outcomes for tumor development in different scenarios, such as senescence in normal and cancer cells.

## Future directions

Based on recent studies there is a need to understand underlying epigenetic mechanisms depending on: (a) the mode of senescence trigger, for example RS, OIS, chemotherapy, reactive oxygen species; (b) the cell/tissue-type specific context; (c) whether or not the cells are normal or tumor. In addition to understanding the basic biology, such studies may provide markers to discriminate *in vivo* senescent, aging and transformed cells, which will have important implications for cancer therapy and prevention.

## References

[1] Campisi J (2013). Annu Rev Physiol.

[2] Narita M (2003). Cell.

[3] Baylin SB, Jones PA (2011). Nat Rev Cancer.

[4] Cruickshanks HA (2013). Nat Cell Biol.

[5] Xie W (2018). Cancer Cell.

[6] Rakyan VK (2010). Genome Res.

[7] Yu Y (2018). Cancer Cell.

[8] Milanovic M (2018). Nature.

